# A pictorial key to differentiate the recently detected exotic *Haemaphysalislongicornis* Neumann, 1901 (Acari, Ixodidae) from native congeners in North America

**DOI:** 10.3897/zookeys.818.30448

**Published:** 2019-01-23

**Authors:** Andrea M. Egizi, Richard G. Robbins, Lorenza Beati, Santiago Nava, Colleen R. vans, James L. Occi, Dina M. Fonseca

**Affiliations:** 1 Tick-borne Diseases Laboratory, Monmouth County Mosquito Control Division, 1901 Wayside Road, Tinton Falls, NJ, 07724-4451, USA; 2 Center for Vector Biology, Department of Entomology, Rutgers University, 180 Jones Avenue, New Brunswick, NJ, 08901-8536, USA; 3 Walter Reed Biosystematics Unit, Department of Entomology, Smithsonian Institution, MSC, MRC 534, 4210 Silver Hill Road, Suitland, MD, 20746-2863, USA; 4 US National Tick Collection, Institute for Coastal Plain Science, Georgia Southern University, 202 Georgia Avenue, Statesboro, GA, 30460, USA; 5 Instituto Nacional de Tecnología Agropecuaria, Estación Experimental Agropecuaria Rafaela, Consejo Nacional de Investigaciones Científicas y Técnicas, CC 22, CP 2300 Rafaela, Santa Fe, Argentina; 6 Andrea M. Egizi

**Keywords:** Asian longhorned tick, haemaphysaline fauna, dichotomous key, scanning electron microscopy, invasive species

## Abstract

Until recently, only two haemaphysaline species, *Haemaphysalischordeilis* (Packard, 1869) and *Haemaphysalisleporispalustris* (Packard, 1869), were known to occur in the United States, and neither was considered to be of significant medical or veterinary importance. In 2017–2018 established populations of the Asian longhorned tick, *Haemaphysalislongicornis* Neumann, 1901, were detected in the eastern US for the first time. *Haemaphysalislongicornis* has the potential to be a significant threat to human and animal health, and the urgent need to determine the full extent of its distribution and host range requires availability of a straightforward and practical guide to differentiate it from native species. We created a pictorial dichotomous key to all stages of *Haemaphysalis* spp. known to occur in North America with scanning electron photomicrographs of all *H.longicornis* life stages, including rarely seen males, to aid researchers in differentiating these species. The largely Neotropical species *Haemaphysalisjuxtakochi* Cooley, 1946, with established populations in Mexico and sporadic detections in the US on migrating birds is also included.

## Introduction

Only two native species of *Haemaphysalis* ticks are known to occur in the United States: the rabbit tick, *Haemaphysalisleporispalustris* (Packard, 1869) and the bird tick, *Haemaphysalischordeilis* (Packard, 1869) (Keirans and Litwak 1989). *Haemaphysalisleporispalustris* is common and widespread in North America, and is frequently collected from lagomorphs (rabbits and hares) (Bishopp and Trembley 1945). Its full distribution extends from Alaska and Canada southward to Argentina (Kohls 1960, Guglielmone et al. 2003). The agents of tularemia (*Francisellatularensis*) and of Rocky Mountain spotted fever (*Rickettsiarickettsii*) have been isolated from this tick (Eremeeva et al. 2018) although its role, if any, as a vector of human disease appears to be minor. *Haemaphysalischordeilis* is far less often collected but nonetheless assumed to have a broad distribution in North America, based on sporadic avian records spanning the US and southern Canada (Bishopp and Trembley 1945, Kohls 1960, Lindquist et al. 2016). Because these two species have historically been considered specialists on rabbits and birds, respectively, and are not significant pests of humans or domestic animals, relatively little attention has been paid to their ecology and geographical distribution.

In 1993, a single specimen of the Central/South American species *Haemaphysalisjuxtakochi* Cooley, 1946 (*Haemaphysaliskochi* Aragão, 1908 is a junior synonym) was detected in Ohio, USA, on a white-tailed deer at a deer-checking station (Keirans and Restifo 1993). While the current northern limit of this species’ distribution appears to be Mexico, immatures may occasionally be brought into the US by northward migrating birds (Mukherjee et al. 2014). At present there is no indication that such encounters are common or that the species has become established in the US. Adult *H.juxtakochi* are chiefly parasites of deer (Kohls 1960, Guglielmone et al. 2005) and may be able to transmit some species of *Rickettsia* (Souza et al. 2018).

In 2017, established populations of the East Asian/Australasian species *Haemaphysalislongicornis* Neumann, 1901, were discovered in New Jersey (Rainey et al. 2018) and subsequently throughout a large part of the eastern US, including Arkansas, Connecticut, Maryland, New York, North Carolina, Pennsylvania, Virginia, and West Virginia (Beard et al. 2018). This species, native to East Asia and invasive in Australia/New Zealand, is associated with disease transmission to humans in the former region (e.g., Zhuang et al. 2018) and is a serious pest of livestock in the latter (Heath 2016). Invasive populations of this species appear to be parthenogenetic, which may facilitate their establishment and spread (Heath 2013). As a result, there is now much concern over this species’ potential effect on human and animal health in the United States, and studies are under way to clarify its current geographic range and preferred host species.

In order to study potential impacts of *H.longicornis* on North America, a critical first step is being able to differentiate this species from co-occurring *Haemaphysalis* spp. Here we present scanning electron photomicrographs of all stages of *H.longicornis*, as well as a simple, usable dichotomous key to differentiate the four *Haemaphysalis* species that may be encountered in North America: *H.leporispalustris*, *H.chordeilis*, *H.longicornis*, and *H.juxtakochi*. While the rarity of *H.juxtakochi* detections in the US does not suggest that this species will often be sympatric with *H.longicornis*, we feel it is important to include it in our key for three reasons: (1) climate change is predicted to alter the distribution of many tick species (Ostfeld and Brunner 2015), therefore the distribution of *H.juxtakochi* may one day shift north of Mexico; (2) unlike some of the exotic species imported by birds (Mukherjee et al. 2014), *H.juxtakochi* could easily find suitable host species in the US; and (3) as it continues its invasion of North America, *H.longicornis* may eventually be collected farther south, coinciding with *H.juxtakochi*’s existing range.

## Materials and methods

### Scanning electron microscopy (SEM)

**Figure 1.**
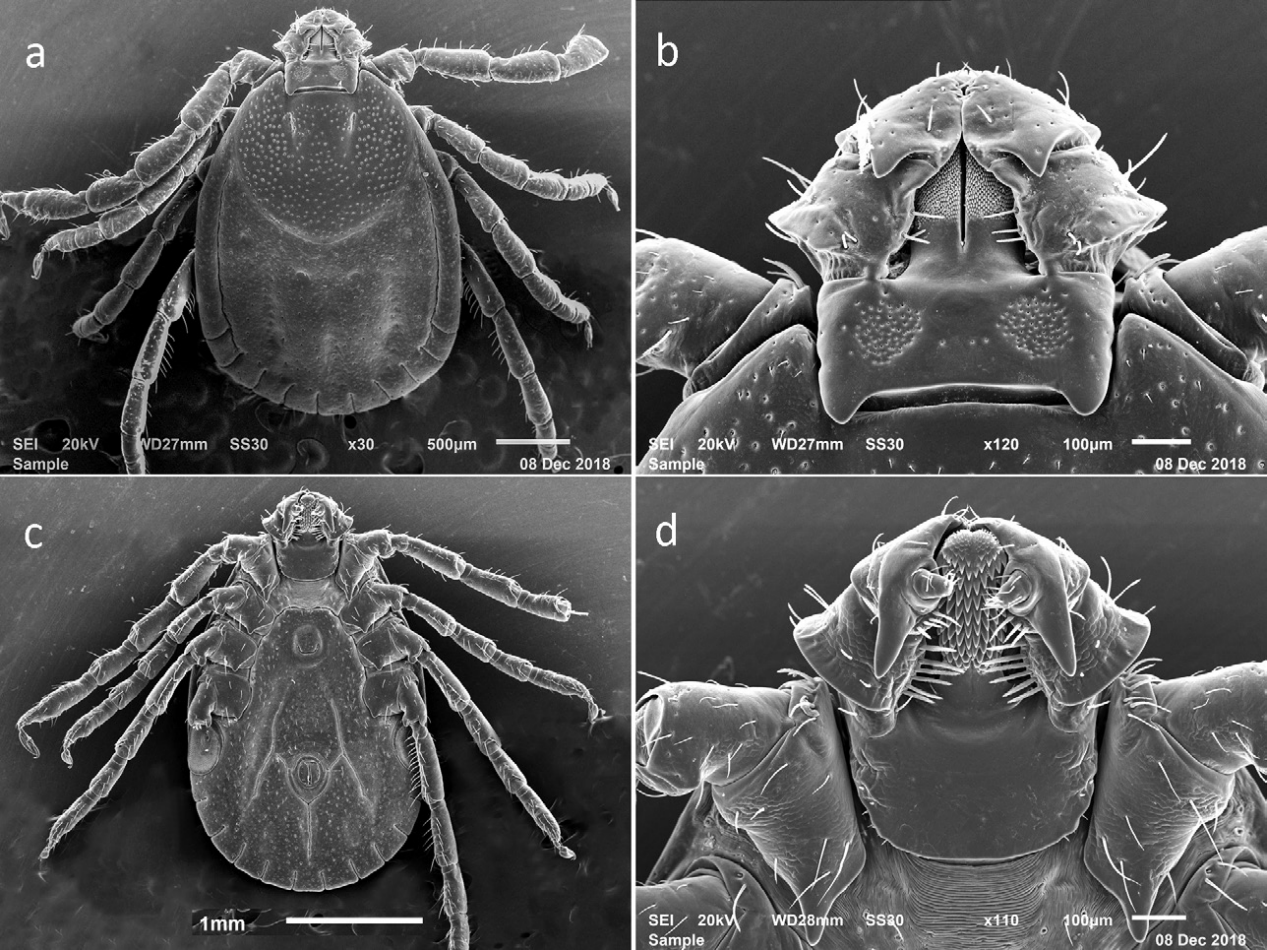
SEM photos of female *H.longicornis* from a colony started with specimens from Jeju-teukbyeoljachido, Republic of Korea (Accession # RML48803) **a** dorsal full body **b** dorsal capitulum **c** ventral full body **d** ventral capitulum.

**Figure 2.**
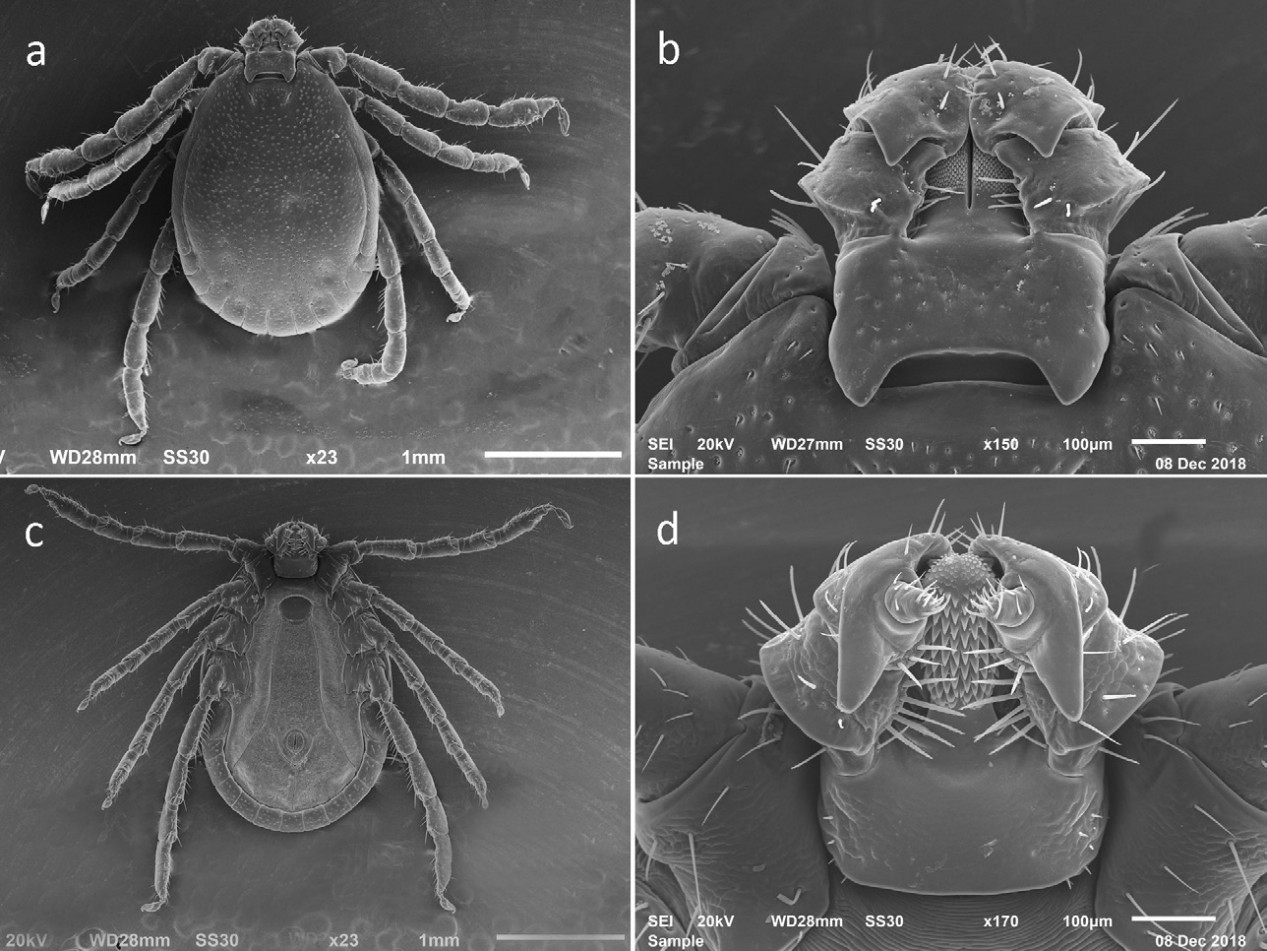
SEM photos of male *H.longicornis* from a colony started with specimens from Jeju-teukbyeoljachido, Republic of Korea. (Accession # RML48803) **a** dorsal full body **b** dorsal capitulum **c** ventral full body **d** ventral capitulum.

**Figure 3.**
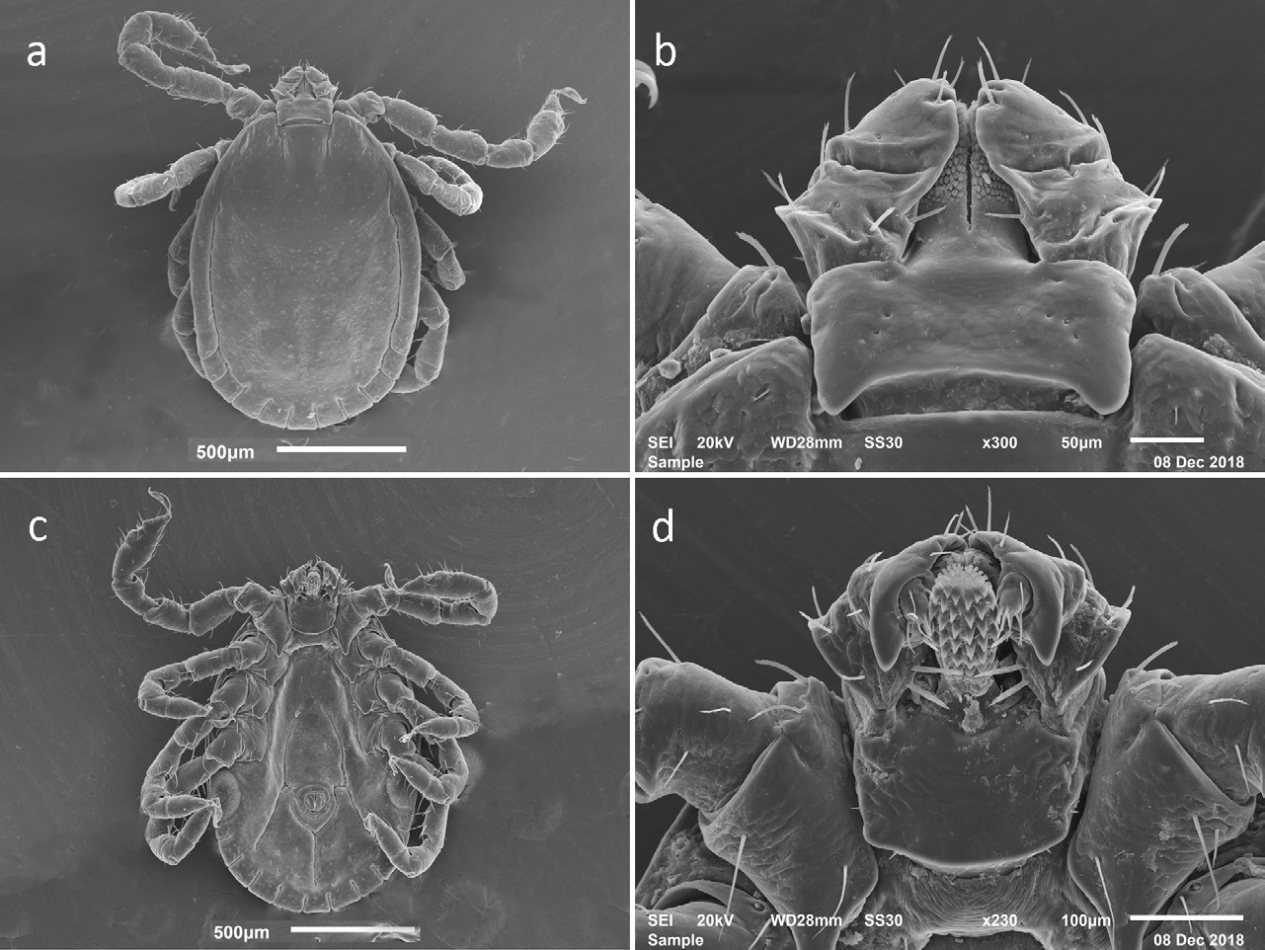
SEM photos of nymphal *H.longicornis* from a colony started with specimens from Jeju-teukbyeoljachido, Republic of Korea (Accession # RML48803). **a** dorsal full body **b** dorsal capitulum **c** ventral full body **d** ventral capitulum.

**Figure 4.**
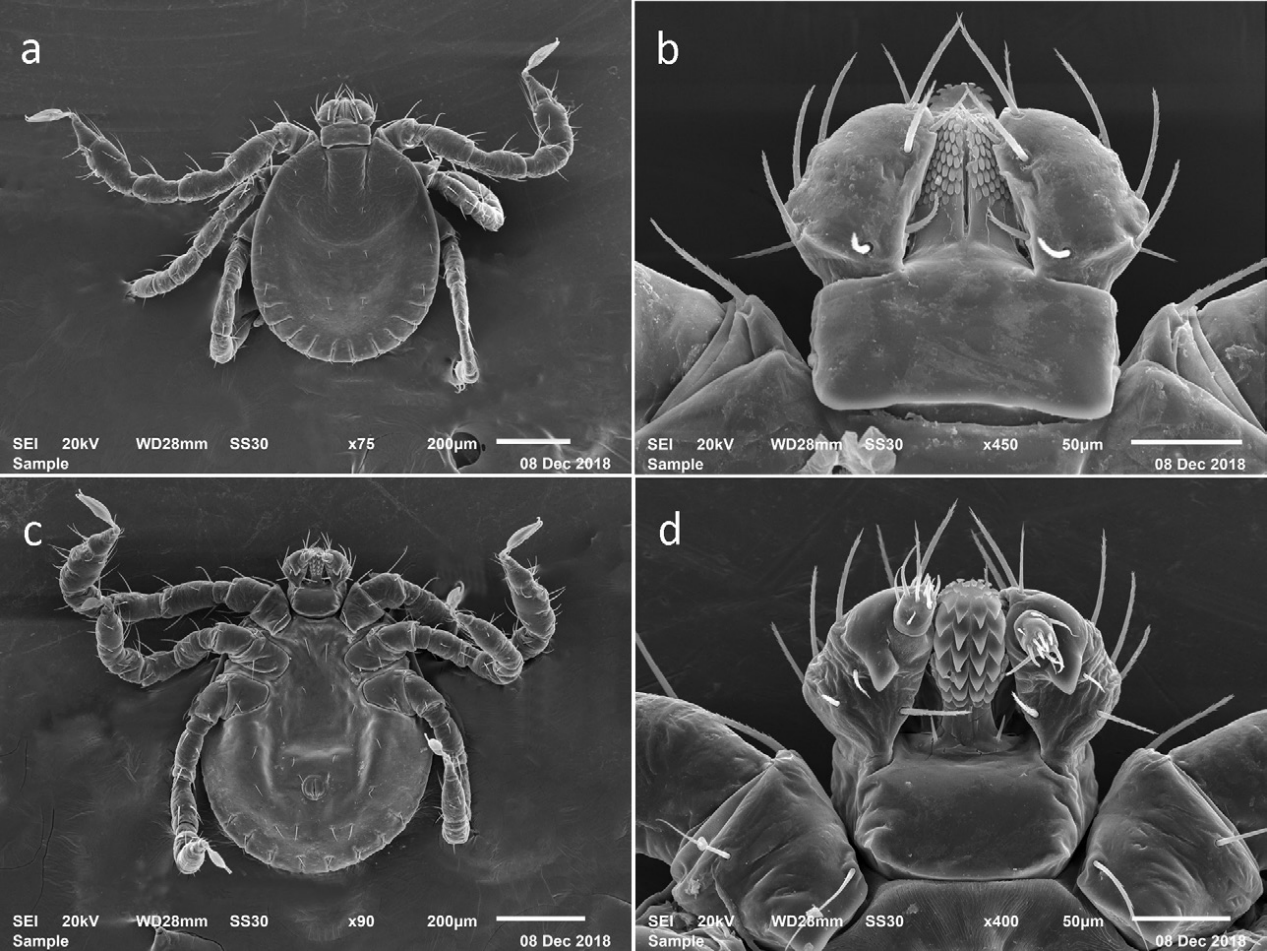
SEM photos of larval *H.longicornis* from a colony started with specimens from Queensland, Australia (Accession # RML58949). **a** dorsal full body **b** dorsal capitulum **c** ventral full body **d** ventral capitulum.

Specimens of *H.longicornis* were obtained from US National Tick Collection archives for imaging. Males, females, and nymphs were sent from a laboratory colony started with specimens collected in Jeju-teukbyeoljachido, Republic of Korea (Accession # RML48803). Larvae originated from a colony started with specimens from Queensland, Australia (Accession # RML58949). Specimens were coated with gold and imaged with a JEOL JSM-6610LV scanning electron microscope (JEOL USA, Inc., Peabody, MA) (Figs 1–4). Larval and nymphal *H.juxtakochi* were collected by flagging in Guanacaste National Park, Costa Rica, and imaged in the same manner (Accession # USNMENT 986092).

Additional photomicrographs of *H.juxtakochi* (adult), *H.leporispalustris* (all stages) and *H.chordeilis* (all stages) were obtained from the US National Tick Collection archives (http://www.discoverlife.org).

### Pictorial dichotomous key

A literature search was conducted and key characters useful for distinguishing the four species were gleaned from the following: Cooley (1946), Kohls (1960), Clifford et al. (1961), Fairchild (1966), Hoogstraal et al. (1968), and Hoogstraal and Kim (1985). Of note, characters chosen to distinguish adult stages are present in both males and females of their respective species.

#### Key to *Haemaphysalis* spp. of North America

Adults (Fig. 5)

**Figure 5.**
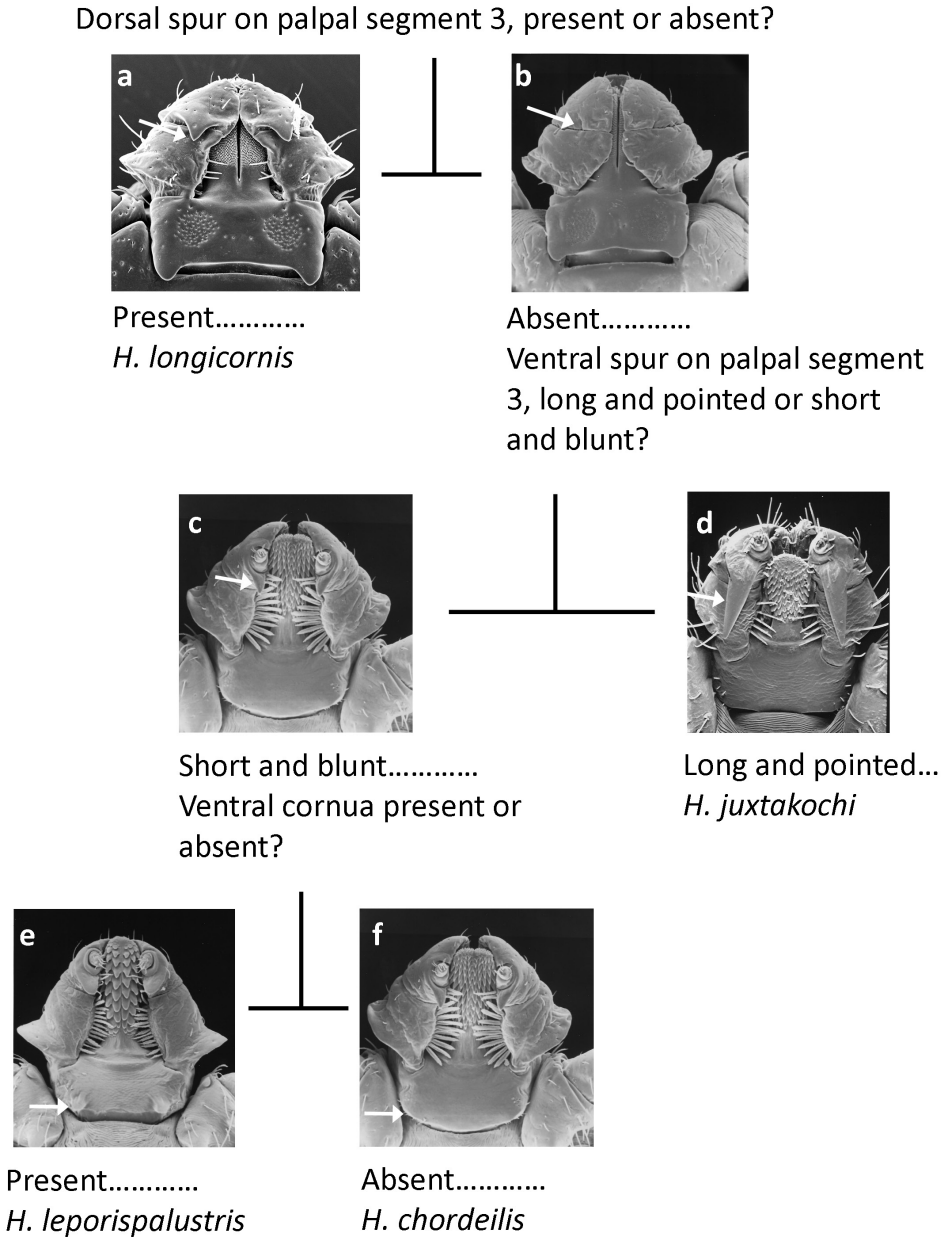
Pictorial key to adults of *Haemaphysalis* spp. occurring in North America.

**Table:** 

1	Palpal segment 3 dorsally with prominent retrograde spur (Fig. 5a)	**Haemaphysalis (Kaiseriana) longicornis Neumann, 1901**
–	Palpal segment 3 without dorsal spur (Fig. 5b)	**2**
2	Palpal segment 3 ventrally with long, slender, retrograde spur extending at least to middle of segment 2 (Fig. 5d)	**Haemaphysalis (Gonixodes) juxtakochi Cooley, 1946**
–	Palpal segment 3 ventrally with short spur, not reaching segment 2 (Fig. 5c)	**3**
3	Basis capituli ventrally with cornua at postero-lateral margins; dental formula 3/3 (Fig. 5e)	**Haemaphysalis (Gonixodes) leporispalustris (Packard, 1869)**
–	Basis capituli ventrally without cornua; dental formula 5/5 (Fig. 5f)	**Haemaphysalis (Aboimisalis) chordeilis (Packard, 1869)**

Nymphs (Fig. 6)

**Figure 6.**
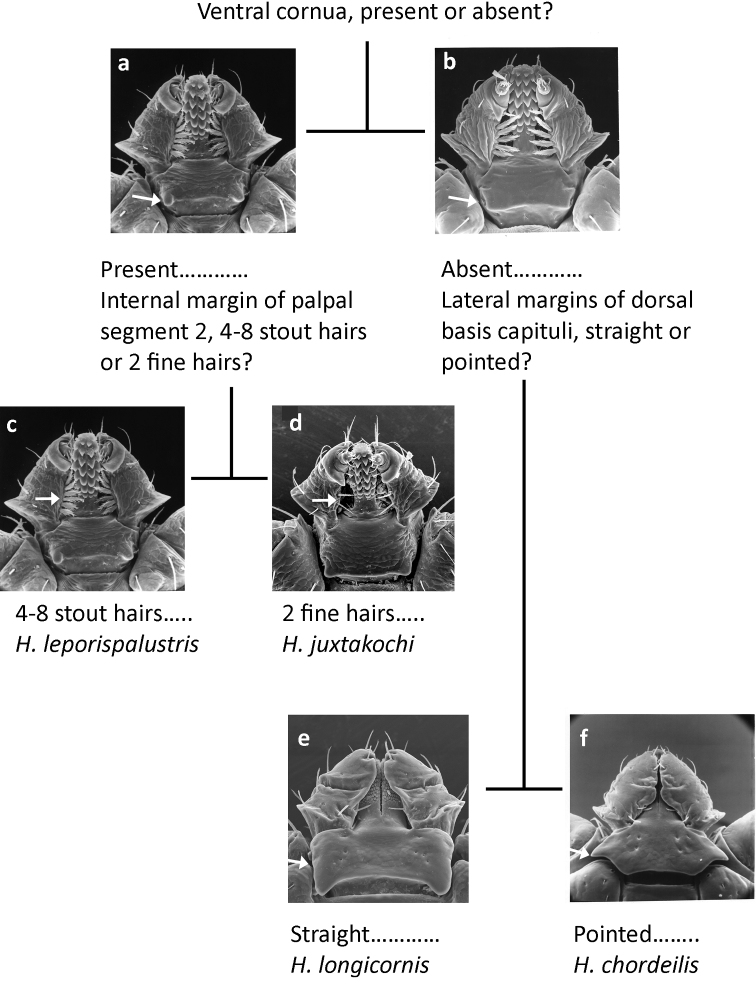
Pictorial key to nymphs of *Haemaphysalis* spp. occurring in North America.

**Table:** 

1	Basis capituli ventrally with cornua at postero-lateral margins (Fig. 6a)	**2**
–	Basis capituli ventrally without cornua at postero-lateral margins (Fig. 6b)	**3**
2	Palpal segment 2 ventrally with 4–8 stout hairs along internal margin; palpal segment 3 ventrally with a short, blunt spur, not reaching anterior margin of segment 2 (Fig. 6c)	**Haemaphysalis (Gonixodes) leporispalustris (Packard, 1869)**
–	Palpal segment 2 ventrally with 2 fine hairs along internal margin; palpal segment 3 ventrally with a longer, sharp, retrograde spur, extending to or beyond anterior margin of segment 2 (Fig. 6d)	**Haemaphysalis (Gonixodes) juxtakochi Cooley, 1946**
3	Dorsally, lateral margins of basis capituli straight (Fig. 6e); hypostomal dental formula 3/3	**Haemaphysalis (Kaiseriana) longicornis Neumann, 1901**
–	Dorsally, lateral margins of basis capituli pointed (Fig. 6f); hypostomal dental formula 2/2	**Haemaphysalis (Aboimisalis) chordeilis (Packard, 1869)**

Larvae (Fig. 7)

**Figure 7.**
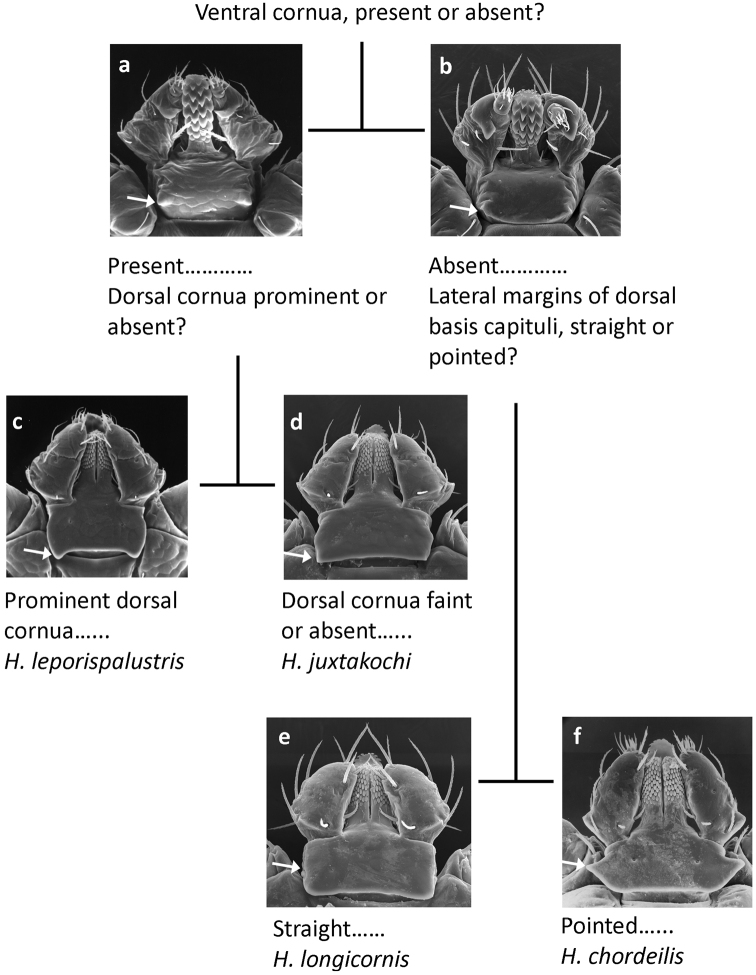
Pictorial key to larvae of *Haemaphysalis* spp. occurring in North America.

**Table:** 

1	Basis capituli ventrally with cornua at postero-lateral margins (Fig. 7a)	**2**
–	Basis capituli ventrally without cornua at postero-lateral margins (Fig. 7b)	**3**
2	Basis capituli dorsally with prominent posteriorly directed cornua (Fig. 7c)	**Haemaphysalis (Gonixodes) leporispalustris (Packard, 1869)**
–	Basis capituli dorsally with cornua faint or absent (Fig. 7d)	**Haemaphysalis (Gonixodes) juxtakochi Cooley, 1946**
3	Dorsally, lateral margins of basis capituli straight (Fig. 7e)	**Haemaphysalis (Kaiseriana) longicornis Neumann, 1901**
–	Dorsally, lateral margins of basis capituli pointed (Fig. 7f)	**Haemaphysalis (Aboimisalis) chordeilis (Packard, 1869)**


## Conclusions

This key enables researchers to distinguish the four species of *Haemaphysalis* that may be encountered in North America in all life stages. Previously, readers would have had to peruse keys from several distinct parts of the world in order to compare the morphology of these four species, e.g. US keys containing *H.chordeilis* (Furman and Loomis 1984, Keirans and Litwak 1989); Old World keys with *H.longicornis*, including Japan (Yamaguti et al. 1971) and Australia (Roberts 1970, Barker and Walker 2014); and Central and South American keys for *H.juxtakochi* (Fairchild et al. 1966, Nava et al. 2017).

The ability to easily distinguish these four species will contribute to ongoing efforts to map the distribution of *Haemaphysalislongicornis* in North America and understand the potential risks posed by this recently discovered exotic tick species (Rainey et al. 2018). This tool will also help to improve our understanding of the biology and ecology of native *Haemaphysalis* spp., which have been relatively poorly studied compared to other native ixodids, and will promote the early detection of any northward expansions of *H.juxtakochi*. In this manner we can capitalize on the interest generated by the arrival of *H.longicornis* to augment our understanding of the existing New World haemaphysaline fauna.

However, as *Haemaphysalis* is the second largest genus in the tick family Ixodidae (so-called hard ticks), with over 160 additional species in the Old World (Petney et al. 2007, Guglielmone et al. 2014), including important disease vectors (de la Fuente et al. 2008), careful monitoring to detect the potential arrival of other members of this genus is encouraged. Should additional *Haemaphysalis* species establish themselves in North America, this key will require revision.

## References

[B1] Barker SC Walker AR (2014) Ticks of Australia: The species that infest domestic animals and humans. Zootaxa 3816: 1–144. 10.11646/zootaxa.3816.1.1 24943801

[B2] Beard CB Occi J Bonilla DL Egizi AM Fonseca DM Mertins JW Backenson BP Bajwa WI Barbarin AM Bertone MA Brown J Connally NP Connell ND Eisen RJ Falco RC James AM Krell RK Lahmers K Lewis N Little SE Neault M Pérez de León AA Randall AR Ruder MG Saleh MN Schappach BL Schroeder BA Seraphin LL Wehtje M Wormser GP Yabsley MJ Halperin W (2018) Multistate Infestation with the Exotic Disease–Vector Tick *Haemaphysalislongicornis* – United States, August 2017 – September 2018. MMWR Morbidity and Mortality Weekly Report 67: 1310–1313. 30496158 10.15585/mmwr.mm6747a3 PMC6276380

[B3] Bishopp F Trembley L (1945) Distribution and hosts of certain North American ticks. Journal of Parasitology 31: 1–41.

[B4] Clifford CM Anastos G Elbl A (1961) The larval ixodid ticks of the eastern United States (Acarina-Ixodidae). Miscellaneous Publications of the Entomological Society of America 2: 213–237.

[B5] Cooley RA (1946) The genera *Boophilus*, *Rhipicephalus*, and *Haemaphysalis* (Ixodidae) of the New World. National Institute of Health Bulletin No. 187, 54 pp.

[B6] de la Fuente J Estrada-Peña A Venzal JM Kocan KM Soneshine DE (2008) Overview: Ticks as vectors of pathogens that cause disease in humans and animals. Frontiers in Bioscience 13: 6938–6946. 18508706 10.2741/3200

[B7] Eremeeva ME Weiner LM Zambrano ML Dasch GA Hu R Vilcins I Castro MB Bonilla DL Padgett KA (2018) Detection and characterization of a novel spotted fever group *Rickettsia* genotype in *Haemaphysalisleporispalustris* from California, USA. Ticks and Tick-borne Diseases 9: 814–818. 29545107 10.1016/j.ttbdis.2018.02.023

[B8] Fairchild G Kohls G Tipton V (1966) The Ticks of Panama (Acarina: Ixodidae). In: Wenzel RL Tipton VJ (Eds) Ectoparasites of Panama.Field Museum of Natural History, Chicago, Illinois, 167–219.

[B9] Furman DP Loomis EC (1984) The Ticks of California (Acari:Ixodida). Bulletin of the California Insect Survey Vol. 25, University of California Press, 239 pp.

[B10] Guglielmone AA Estrada-Peña A Keirans JE Robbins RG (2003) Ticks (Acari: Ixodida) of the Neotropical Zoogeographic Region. Special publication of the International Consortium on Ticks and Tick-borne Diseases. Atalanta, Houten, 173 pp.

[B11] Guglielmone AA Romero J Venzal JM Nava S Mangold AJ Villavicencio J (2005) First record of *Haemaphysalisjuxtakochi* Cooley, 1946 (Acari: Ixodidae) from Peru. Systematic & Applied Acarology 10: 33–35.

[B12] Guglielmone AA Robbins RG Apanaskevich DA Petney TN Estrada-Peña A Horak IG (2014) The Hard Ticks of the World (Acari: Ixodida: Ixodidae). Springer, Dordrecht, 738 pp.

[B13] Heath ACG (2013) Implications for New Zealand of potentially invasive ticks sympatric with *Haemaphysalislongicornis* Neumann, 1901 (Acari: Ixodidae). Systematic & Applied Acarology 18: 1–26.

[B14] Heath ACG (2016) Biology, ecology and distribution of the tick, *Haemaphysalislongicornis* Neumann (Acari: Ixodidae) in New Zealand. New Zealand Veterinary Journal 64: 10–20. 10.1080/00480169.2015.1035769 25849758

[B15] Hoogstraal H Kim KC (1985) Tick and mammal coevolution, with emphasis on *Haemaphysalis*. In: Kim KC (Ed.) Coevolution of Parasitic Arthropods and Mammals.John Wiley & Sons, New York, 505–568.

[B16] Hoogstraal H Roberts FHS Kohls GM Tipton VJ (1968) Review of Haemaphysalis (Kaiseriana) longicornis Neumann (resurrected) of Australia, New Zealand, New Caledonia, Fiji, Japan, Korea, and northeastern China and USSR, and its parthenogenetic and bisexual populations (Ixodoidea, Ixodidae). Journal of Parasitology 54: 1197–1213. 5757695

[B17] Keirans JE Litwak TR (1989) Pictorial key to the adults of hard ticks, family Ixodidae (Ixodida: Ixodoidea), east of the Mississippi River. Journal of Medical Entomology 26: 435–448. 10.1093/jmedent/26.5.435 2795615

[B18] Keirans JE Restifo RA (1993) *Haemaphysalisjuxtakochi* Cooley (Acari: Ixodidae), a Neotropical tick species, found in Ohio. Journal of Medical Entomology 30: 1074–1075. 8271252 10.1093/jmedent/30.6.1074

[B19] Kohls GM (1960) Records and new synonymy of New World *Haemaphysalis* ticks, with descriptions of the nymph and larva of *H.juxtakochi* Cooley. Journal of Parasitology 46: 355–361. 14410554

[B20] Lindquist EE Galloway TD Artsob H Lindsay LR Drebot M Wood H Robbins RG (2016) A Handbook to the Ticks of Canada (Ixodida: Ixodidae, Argasidae). Biological Survey of Canada Monograph No. 7, Ottawa, 317 pp.

[B21] Mukherjee N Beati L Sellers M Burton L Adamson S Robbins RG Moore F Karim S (2014) Importation of exotic ticks and tick-borne spotted fever group rickettsiae into the United States by migrating songbirds. Ticks and Tick-borne Diseases 5: 127–134. 10.1016/j.ttbdis.2013.09.009 24252263 PMC3946858

[B22] Nava S Venzal JM González-Acuña D Martins TF Guglielmone AA (2017) Ticks of the Southern Cone of America: Diagnosis, Distribution, and Hosts with Taxonomy, Ecology and Sanitary Importance. Academic Press, 372 pp.

[B23] Ostfeld RS Brunner JL (2015) Climate change and *Ixodes* tick-borne diseases of humans. Philosophical Transactions of the Royal Society B 370: 20140051. 10.1098/rstb.2014.0051 PMC4342967 25688022

[B24] Petney TN Kolonin GV Robbins RG (2007) Southeast Asian ticks (Acari: Ixodida): a historical perspective. Parasitology Research 101(Suppl. 2): S201–205. 10.1007/s00436-007-0687-4 17823829

[B25] Rainey T Occi JL Robbins RG Egizi A (2018) Discovery of *Haemaphysalislongicornis* (Ixodida: Ixodidae) parasitizing a sheep in New Jersey, United States. Journal of Medical Entomology 55: 757–759. 10.1093/jme/tjy006 29471482

[B26] Roberts FHS (1970) Australian Ticks. Commonwealth Scientific and Industrial Research Organization, Melbourne, 267 pp.

[B27] Souza U Dall’Agnol B Michel T Webster A Weck B Doyle R Kasper CB Soares J Martins JR Trigo TC Ott R Jardim MMA Reck J (2018) Molecular survey of *Rickettsia* spp. in the Neotropical deer tick *Haemaphysalisjuxtakochi* from Brazilian Pampa. Parasitology Research. 10.1007/s00436-018-5996-2 29980888

[B28] Yamaguti N Tipton VJ Keegan HL Toshioka S (1971) Ticks of Japan, Korea, and the Ryukyu Islands. Brigham Young University Science Bulletin, Biological Series 15(1): 1–226.

[B29] Zhuang L Sun Y Cui XM Tang F Hu JG Wang LY Cui N Yang ZD Huang DD Zhang XA Liu W Cao WC (2018) Transmission of severe fever with thrombocytopenia syndrome virus by *Haemaphysalislongicornis* ticks, China. Emerging Infectious Diseases 24: 868–871. 10.3201/eid2405.151435 PMC5938789 29664718

